# Mechanisms of autoimmune-mediated paraneoplastic syndromes: immune tolerance and disease pathogenesis

**DOI:** 10.3389/fimmu.2025.1608934

**Published:** 2025-05-09

**Authors:** César Pérez-Bucio, Anish Behere, Nils Landegren

**Affiliations:** Science for Life Laboratory, Department of Medical Biochemistry and Microbiology, Uppsala University, Uppsala, Sweden

**Keywords:** autoimmune-mediated paraneoplastic syndromes, autoantibodies, self-tolerance, tumor microenvironment, immune related adverse events (irAEs)

## Abstract

Paraneoplastic syndromes represent a clinically heterogeneous group of disorders that arise in cancer patients. Although their underlying mechanisms are only partly understood, immune or endocrine mechanisms are believed to play key roles. Autoimmune-mediated paraneoplastic syndromes (AMPS) are typically characterized by the presence of autoantibodies, making their identification important for both AMPS diagnosis and early cancer detection. This review synthesizes emerging insights into the pathogenesis of AMPS, with a particular focus on how genomic instability in cancer cells promotes immune recognition of altered self-proteins. Mechanisms such as ectopic expression, protein modifications (such as isoaspartylation), and gene amplifications can disrupt immune tolerance, leading to autoimmunity. Additionally, chronic inflammation and the formation of tertiary lymphoid structures within the tumor microenvironment contribute to both antitumor immunity and autoimmunity. Immune checkpoint inhibitors (ICIs), have revolutionized cancer treatment by enhancing antitumor immunity, but they can also induce immune-related adverse events (irAEs), some of which mimic AMPS. These irAEs highlight the critical roles of both humoral and cellular immunity in AMPS development. By exploring the relationships between ICI treatment, immune tolerance, and tumor-specific antigens, this review aims to clarify the mechanisms driving AMPS and their dual role in cancer control and immune-mediated disease. Bridging these knowledge gaps may inform the development of novel therapeutic strategies for managing AMPS and in optimizing the use of ICIs in cancer care.

## 1 Introduction

Paraneoplastic syndromes (PS) comprise a diverse group of disorders that affect different organ systems in cancer patients, arising independently of the primary tumor or its metastases. Their clinical manifestations are often distant from the tumor site and are thought to result from humeral factors produced by cancer cells or by immune system reactions. Due to their rarity and clinical heterogeneity, the reported incidence of PS varies widely, ranging from 7% to 33% of cancer patients (1, 2). These syndromes typically manifest within ±3 years of cancer diagnosis and are more frequently identified after cancer is detected (3).

The mechanisms underlying PS are incompletely understood and diverse. Non-autoimmune-mediated PS are often driven by endocrine imbalances (4), such as hyponatremia, hypercalcemia, or Cushing’s syndrome, as well as cytokine dysregulation (5), including neutrophilia, Trousseau’s syndrome, and thrombocytosis. These cases are associated with poor patient outcomes (5). In contrast, autoimmune-mediated paraneoplastic syndromes are linked to better cancer prognoses, suggesting that autoimmune flares are a sign of enhanced antitumor responses.

Autoimmune-mediated paraneoplastic syndromes (AMPS) most commonly affect the central nervous system (CNS) but can also manifest in the peripheral nervous system (PNS), skin, or blood (6). A hallmark of AMPS is the presence of autoantibodies, which have gained attention for their role as disease biomarkers (7). These autoantibodies are important in the diagnosis of paraneoplastic syndromes and may support early cancer detection in this group of patients. For instance, individual autoantibodies, such as p53 (8), HER2 (9), and NY-ESO-1 (10), as well as autoantibody panels, have shown predictive potential for cancer diagnosis (11, 12). However, these biomarkers still fail to meet clinical standards (13). Important observations have been made linking cancer alterations with commonly associated autoantibodies and the development of AMPS (**Table 1**).

**Table 1:** Summary of autoimmune-mediated paraneoplastic syndromes (AMPS).

Manifestations	Syndrome	Underlying Cancers	Associated Autoantibody
Neurological	Lambert–Eaton myasthenic syndrome (14)	SCLC	Anti-VGCC (P/Q-type voltage-gated calcium channel)
	Paraneoplastic cerebellar degeneration (15)	Lung cancer, ovarian cancer, breast carcinoma, Hodgkin’s lymphoma	Anti-Yo, Anti-Hu, Anti-Tr, Anti-Ri
	Sensory Neuronopathies (16)	Various	Anti-Hu, Anti-CRPM5, Anti-CV2, Anti-AGO
	Encephalomyelitis (17)	Various	Anti-Hu
	Limbic encephalitis (18)	SCLC	Anti-Hu, Anti-Ma2
	Brainstem encephalitis (15, 19)	Lung cancer, testicular cancer	Anti-Hu, Anti-Ri, Anti-Ma2, Anti-KLHL11
	Opsoclonus myoclonus ataxia syndrome (20)	Breast carcinoma, ovarian carcinoma, SCLC, neuroblastoma (in children)	Anti-Ri
	Myasthenia Gravis (21)	Thymoma	Anti-AChR
	Anti-NMDA receptor encephalitis (22)	Teratoma	Anti-NMDA receptor
Mucocutaneous	Polymyositis (23)	Non-Hodgkin lymphoma, lung cancer, bladder cancer	Anti-Jo-1
	Acanthosis nigricans (24)	Gastric carcinoma, lung carcinoma, uterine carcinoma	N.A.
	Dermatomyositis (23)	Bronchogenic carcinoma, breast carcinoma, ovarian cancer, pancreatic cancer, stomach cancer, colorectal cancer, non-Hodgkin lymphoma	Anti-Mi-2, Anti-NXP2, Anti-TIF1γ
	Leser-Trélat sign (25)	Various	N.A.
	Necrolytic migratory erythema (26)	Glucagonoma	N.A.
	Sweet’s syndrome (27)	Hematologic malignancies	N.A.
	Leukocytoclastic vasculitis (28)	Leukemia/lymphoma, myelodysplastic syndromes, colon, lung, urologie, multiple myeloma, rhabdomyosarcoma	N.A.
	Paraneoplastic pemphigus (29, 30)	Non-Hodgkin lymphoma, chronic lymphocytic leukemia, thymoma, Castleman disease, follicular dendritic cell sarcoma	Anti-Plakin, Anti-Desmoglein
Rheumatic	Palmar fasciitis and polyarthritis (31)	Ovarian cancer	N.A.
	Sjögren-like syndrome (32)	Non-Hodgkin lymphoma, lung cancer	Anti-Ro/SSA, Anti-La/SSB
	Polymyalgia rheumatica (33)	Leukemia/lymphoma, myelodysplastic syndromes, colon, lung, renal, prostate, breast cancer	N.A.
	Systemic Sclerosis (SSc) (34)	Breast, lung, prostate cancer, melanoma	Anti-RPC-1
	Hypertrophic osteoarthropathy (35)	Lung cancer (adenocarcinoma), mesothelioma	N.A.
Others	Membranous glomerulonephritis (36)	Various	Anti-PLA2R
	Tumor-induced osteomalacia (37)	Hemangiopericytoma, phosphaturic mesenchymal tumor	N.A.
	Stauffer syndrome (38)	Renal cell carcinoma	N.A.
	Cancer-Associated Retinopathy (CAR) (39)	Melanoma, SCLC	Anti-recoverin, Anti-transducin

Despite advances in autoantibody research, the cellular and molecular mechanisms underlying AMPS pathogenesis remain poorly understood. This review aims to summarize the current state of research on AMPS exploring the mechanisms driving autoimmune disorders in cancer. Specifically, it will address how ectopic expression, protein structural alterations, and overexpression of self-proteins may trigger autoimmune responses. Overlapping clinical presentations between AMPS and immune-related adverse events (irAEs) of immune checkpoint inhibitor (ICI) treatment will be discussed, highlighting the need to understand autoimmunity in cancer. Further, the roles of chronic inflammation within the tumor microenvironment (TME) and localized immune tolerance in sustaining these responses will be examined. With this discussion, we aim to bridge existing knowledge gaps and provide a framework to better understand AMPS pathogenesis.

## 2 Protein overexpression, ectopic expression and structure changes drive autoimmunity in cancer

Ectopic expression refers to the production of a self-protein in atypical tissues. Malignant cells frequently exhibit ectopic expression of otherwise tissue-restricted proteins as a consequence of genomic instability. Genomic instability may also trigger missense mutations that may lead to changes in protein structure. Similarly, an antigen may be overexpressed due to chromosome amplifications. In cancer, any of these alterations can induce immune recognition and targeting of malignant cells. In the following AMPS cases, evidence has been provided of how genetic or proteomic alterations can lead to immune recognition and the development of autoimmunity.

### 2.1 Anti-Hu: encephalomyelitis

The Hu protein family comprises RNA-binding proteins primarily expressed in neuronal cells, where they play a role in mRNA stabilization and translation (40). HuD, a member of this family, is known to be expressed in several cancer types (41). Small cell lung cancer (SCLC) patients with paraneoplastic sensory neuropathy or encephalomyelitis present high anti-Hu antibody titers (42). These antibodies target tumor-expressed Hu proteins, which would otherwise be restricted to neuronal cells. Interestingly, while all SCLC patients with neurological AMPS are anti-Hu positive, 17% of all SCLC patients, irrespective of paraneoplastic presentation, harbor detectable anti-Hu titers (43).

Changes in protein structure can occur after protein folding, contributing to the development of autoimmunity. HuD (also known as ELAVL4) commonly undergoes isoaspartylation, a modification that can impair its physiological activity. In the healthy CNS, PIMT (peptidyl-isomerase methyltransferase) repairs this damage, restoring HuD to its native, functional form (44). However, because PIMT expression is restricted to certain tissues (45), HuD-expressing tumors accumulate isoaspartylated HuD. This ectopically expressed protein variant can be recognized as a non-self-antigen, triggering an immune response in patients with HuD-expressing cancers. In rare cases, the development of anti-HuD autoantibodies may lead to cross-reactivity and recognition of the wild-type HuD protein in the CNS (46).

Pulido M. and colleagues tested serum from seven anti-HuD positive SCLC patients, four of whom had paraneoplastic syndromes against both native and isoaspartylated HuD. All seven samples reacted with both protein isoforms, suggesting not only immune stimulation of isoaspartylated HuD but also cross-reactivity toward native HuD (46). This cross-reactivity explains the paraneoplastic presentation in some of these patients. Isoaspartyl post-translational modifications has been shown to cause immunogenicity and break self-tolerance before (47).

### 2.2 Anti-recoverin: cancer-associated retinopathy

Cancer-associated retinopathy (CAR) has been observed across various cancer types, predominantly in SCLC and breast cancer. CAR is characterized by progressive sight loss due to autoimmune targeting of retinal proteins. The rarity of this syndrome has made it difficult to reliably identify the underlying autoantibody, although markers against specific retinal proteins like transducin and recoverin have been reported (48, 49).

Sera from 143 cancer patients (99 SCLC and 44 non-small cell lung cancer [NSCLC]) was tested for anti-recoverin antibodies by immunoblotting. Anti-recoverin positivity was observed in 15% and 20% of SCLC and NSCLC cases, respectively. No healthy controls showed anti-recoverin positivity (50). Tumor samples were available from 44 SCLC and 40 NSCLC patients. Recoverin expression was detected by immunohistochemistry in 68% of SCLC and 85% of NSCLC patients (50). All symptomatic CAR-SCLC patients test positive for anti-recoverin autoantibodies, likely due to ectopic expression of retinal proteins (51). A clinical report of a uterine carcinosarcoma patient also revealed serum positivity for anti-recoverin antibodies. Postmortem immunofluorescence analysis detected recoverin expression in the tumor cells (52).

### 2.3 Anti-VGCC: paraneoplastic LEMS

SCLC cells can aberrantly express voltage-gated calcium channels (VGCCs), typically found in presynaptic nerve terminals of the CNS and at neuromuscular junctions (53). The autoimmune disorder Lambert–Eaton myasthenic syndrome (LEMS) arises from the production of anti-P/Q-type VGCC autoantibodies, leading to muscle weakness, dry mouth, and neurodegenerative features (54). LEMS is a paraneoplastic syndrome in approximately 62% of cases (55), with all SCLC-LEMS patients exhibiting anti-P/Q-type VGCC autoantibodies (56), and 25% of them presenting neurological symptoms in addition to the predominant muscle weakness (57).

Not all anti-VGCC positive patients develop LEMS. One study reported that half of the patients (5 out of 10) with anti-VGCC autoantibodies did not exhibit any symptoms of LEMS or neurological damage (58).

### 2.4 Anti-NMDAR: encephalitis

Another autoimmune disorder with neurological symptoms is anti-N-methyl-D-aspartate receptor (NMDAR) encephalitis, which involves the disruption of a key receptor for excitatory neurotransmission specific to the CNS (59). NMDAR is often expressed in ovarian teratomas (OT), and approximately 37% of anti-NMDAR encephalitis cases are associated with ovarian teratomas, where the encephalitis resolves after tumor resection (60). Further, reports have shown that in these cases, the antibodies targeting NMDAR are generated within the tumor tissue (61). Moreover, patients with ovarian teratomas but without neurological symptoms do not exhibit anti-NMDAR antibodies, supporting their association with neurological complications (62–64).

### 2.5 Anti-RPC1: systemic sclerosis

Anti-RNA polymerase III subunit C (RPC1) autoantibodies are commonly found in patients with systemic sclerosis (SSc) (65). A study of eight RPC+ paraneoplastic SSc patients with different forms of cancer revealed that the tumors in five of these patients exhibited genetic alterations in the *POLR3A* locus. Three of them had a missense mutation, and all five presented with loss of heterozygosity. Every patient harbored at least one genetic alteration, strongly suggesting that these genetic changes triggered an immune response against both the cancer-expressed and the wild-type RNA polymerase protein (66).

### 2.6 Anti-Yo: cerebellar degeneration

Cerebellar degeneration-related protein 2 (CDR2) and CDR2-like (CDR2L), are neuronal cell proteins typically expressed in Purkinje cells (referred to as Yo antigens) (67) and have been implicated in paraneoplastic cerebellar degeneration. Anti-Yo autoantibody titers were found in a cohort of patients with paraneoplastic cerebellar damage due to ovarian carcinoma. In this group, 17 of 17 tumor samples tested had at least one genetic alteration. Specifically, 59% of cases exhibited a chromosome 17q gain, where CDR2L and CDR2 are located, and 65% of cases had mutations in either or both *CDR2L*/*CDR2* genes (68).

Similarly, a study of 29 anti-Yo positive breast carcinoma patients revealed that all had at least one genetic alteration such as mutations (62.5%), amplifications (61.5%), or 17q gains (38.4%) in the *CDR2L*/*CDR2* genes, which also contributed to paraneoplastic cerebellar degeneration (69). An interesting observation discussed by the authors is that the tumors were highly infiltrated by B and T cells and tended to metastasize early. Not only showing strong anti-tumor responses but also a high tumor invasiveness in this AMPS cohort (69).

### 2.7 Anti-AChR: myasthenia gravis

Myasthenia Gravis (MG) is an autoimmune disorder that is tightly linked to neoplasia, particularly thymoma. It is estimated that 30% to 40% of thymoma patients develop MG (70, 71). Unlike many cancer-associated autoimmune syndromes, MG is not associated with a favorable prognosis in thymoma patients (72). MG is characterized by progressive muscle weakness that worsens with physical movement, driven by autoantibody activity against neuromuscular-associated proteins. The most common MG autoantibody is anti-acetylcholine receptor (AChR) (85% of MG cases (73)), a protein responsible for signal transmission at the neuromuscular junction (74).

MG-associated thymoma and non-paraneoplastic thymoma tumors were compared by bulk RNA sequencing on two independent studies (75, 76). Both results showed AChR overexpression in MG-associated thymoma samples - 3 and 1.07 fold increase respectively - initially suggesting that protein overexpression may drive autoantibody production. Interestingly, the *NEFM* (Neurofilament Medium Chain) gene coding for a neuronal protein NEFM was found to be highly expressed (30-fold (75) and >100-fold (76)) in MG-associated thymoma cases. Schultz et al. (1999) showed that NEFM contains a linear epitope that closely resembles an AChR antigen, and that NEFM-specific antibodies could recognize the AChR -subunit, suggesting that anti-AChR activity is promoted by immunity against NEFM (77). Similar epitopes in thymoma MG samples that may trigger cross-reacting antibodies against titin and RYR have also been proposed (75).

## 3 Immune checkpoint inhibitors and immune-related adverse events

In the previous section, we discussed cases of AMPS that arise independently of cancer treatment. Immune-modulating treatments such as ICIs can trigger irAEs, some of which share similarities with AMPS. This has become increasingly relevant given the quick adaptation of ICIs into the clinic, driven by their ability to improve overall survival while causing fewer general adverse effects than traditional therapies (78–80).

Immune checkpoints prevent overactivation of cytotoxic T cells, ensuring that self-tolerance is maintained. PD-L1 for instance, dampens T cell cytotoxicity through interaction with PD-1 expressed by T cells. This regulatory function is controlled by immune cells but can also be mediated in immune-privileged tissues such as the lung, liver, and placenta (81). Cancer cells exploit immune checkpoints by upregulating inhibitory signals such as PD-L1 (interacting with PD-1 (82) and CD80 (interacting with CTLA-4 (83)) to evade immune destruction and create an immunosuppressive tumor microenvironment. By inhibiting these pathways, ICIs enhance T cell recognition of cancer cells, but also increase the risk of autoimmunity, particularly in individuals with a history of autoimmune disorders (84, 85).

Several irAEs observed in ICI-treated patients overlap with AMPS; disorders like myositis, encephalitis, SSc, and MG are among the most prevalent overlapping conditions. While clinical similarities suggest that ICI treatment could increase the risk of developing AMPS, there are many contrasting features between them. For example, a systematic review by Buckley et al. (2025) on ICI-related encephalitis, found that only 46.9% of cases were autoantibody-positive (86), different from AMPS cases, where autoantibodies are significantly more frequent (100% in SCLC paraneoplastic encephalitis (42), 75% in general paraneoplastic encephalitides (87)). Similarly, in Hamada et al. (2021), only 67% of ICI-related MG cases were positive for any MG-related autoantibody (88) compared to over 95% in thymoma MG (89). A similar trend of seronegative cases has been noted in ICI-related SSc (90).

While seropositive irAEs seem to be a case where ICIs facilitate development of AMPS, a significant proportion of irAE cases are seronegative and appear to have mechanistic differences, where T cell cytotoxicity is the main driver of autoimmunity. Whether these autoreactive T cells are expanded through antitumor immunity, as observed in AMPS, remains to be explored.

Given the high prevalence of severe irAEs of ICI-treated patients (20% (91)), predictive screening tools could help physicians tailor treatment strategies to individual patients. Biomarkers such as blood counts, autoantibodies, HLA genotype, and microRNA expression profiles have been identified as prognostic and diagnostic tools in autoimmune diseases (92). Several studies suggest that pre-existing autoantibodies may indicate an increased risk of developing irAEs following ICI therapy (93, 94). Screening for autoantibodies before initiating ICI therapy could help classify patients based on their likelihood of developing severe irAEs, enabling safer and personalized treatment approaches.

## 4 Chronic inflammation and localized tolerance breaks

While the presence of autoantibodies is a hallmark of AMPS, not all patients with detectable autoantibodies develop autoimmune symptoms. This discrepancy suggests that additional factors, such as chronic inflammation and localized immune responses within the TME, may determine whether autoimmunity manifests systemically. Understanding these factors is critical for understanding the connection between antitumor immunity and autoimmune pathology.

The disorders summarized earlier demonstrate that AMPS patients harbor autoantibodies capable of inducing immune responses with both cancer-resolving and autoimmune consequences. However, it remains unclear why some patients with autoantibodies benefit from a stronger antitumor response without developing systemic autoimmunity. This observation points to the importance of additional immune triggers beyond humoral activity in the development of AMPS.

Strong antitumor responses, a common feature of AMPS patients, are often accompanied by the presence of tertiary lymphoid structures (TLS) within the chronically inflamed TME (95). TLS are organized immune cell aggregates comprising T cells, B cells, dendritic cells, and macrophages. Functionally resembling secondary lymphoid organs (SLO), TLS promote antigen presentation, B cell priming, and T cell activation (96). In some AMPS cases, the autoimmune response is mediated entirely within the tumor. For example, Al-Diwani et al. (2022) observed that ex vivo cultures of B cells dissociated from ovarian teratomas generated anti-NMDAR antibodies, while B cells from cervical lymph nodes did not produce autoantibodies after tumor resection. This finding supports the idea that autoimmunity is initiated within the tumor-associated TLS (61). Similarly, Mazor et al. (2022) demonstrated that tumor-specific B cells are primed and clonally expanded within the tumor, often originating from naturally occurring self-reactive B cells (97). T cells can also expand and generate antitumor responses within the TLS (98). These findings suggest that TLS facilitate localized antitumor immunity, allowing B and T cells to target self-antigens without eliciting systemic autoimmunity.

This localized immune activity aligns with the observed improved patient prognosis in the presence of TLS (99, 100). Many AMPS cases reviewed here, such as anti-NMDAR encephalitis and anti-Yo cerebellar degeneration, involve highly infiltrated tumors (60, 68, 69). TLS-mediated immunity represents a controlled break in immune tolerance, enabling effective clearance of malignant cells. While circulating autoantibodies are detectable in patient serum, T cells are typically confined to the tumor. A breach in this localization (where T cells migrate from the tumor into circulation) could be the defining trigger for systemic AMPS.

**Figure 1:**
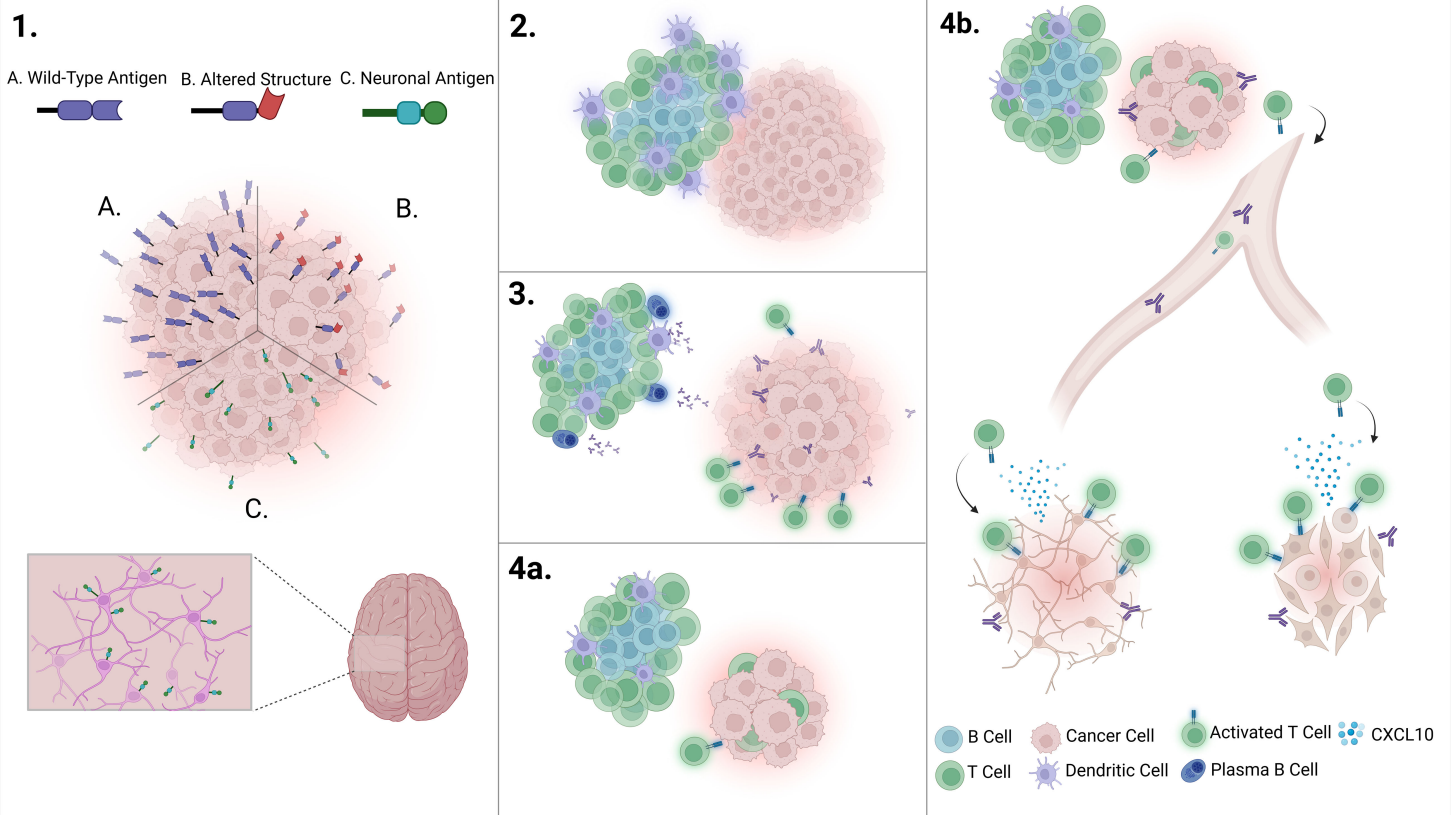
Overview of the pathogenesis of autoimmune-mediated paraneoplastic syndromes. 1. Immune recognition of a tumor occurs through **(A)** antigen overexpression, **(B)** altered protein structure, or **(C)** ectopic antigen expression, initiating an immune response and promoting inflammation. 2. Chronic inflammation within the tumor microenvironment supports the formation of tertiary lymphoid structures (TLS). 3. As the TLS matures, it facilitates the activation of autoreactive tumor-targeting T cells and autoantibody-producing plasma cells, leading to a highly infiltrated tumor. 4a. A robust immune response successfully eliminates the tumor without triggering autoimmune symptoms. While autoantibodies remain detectable in circulation, autoreactive B and T cells remain localized within the tumor microenvironment. 4b. In contrast, remote inflammatory events can recruit T cells, promoting the extravasation of autoreactive T cells. Once in circulation, these T cells can recognize and attack healthy tissues, leading to autoimmunity. Created in BioRender. Perez Bucio, **(C)** (2025) https://BioRender.com/x3cnql9.

Activated T cells tend to accumulate in inflamed tissues due to retention signals such as the chemokine CXCL10 (101). In the absence of systemic inflammation or additional chronic inflammatory events, these T cells are unlikely to enter the bloodstream. However, in cases of invasive cancers with high levels of circulating tumor cells or early metastatic spread, inflammation at the tumor periphery may promote signals that induce T cell migration. This process could extend the anti-self immune response to healthy tissues, contributing to the onset of paraneoplastic syndromes (**Figure 1**).

Recent studies suggest that a humoral immune response alone is insufficient to trigger autoimmune symptoms but rather serves as a marker of broken self-antigen tolerance. For instance, in paraneoplastic SSc (66) and anti-Yo-related paraneoplastic cerebellar degeneration (68, 69), the majority of patients exhibited highly invasive or early metastatic malignancies. This suggests that the expansion of an immune response beyond the tumor, driven by T cell migration, could trigger autoimmune disorders. In contrast, patients with autoantibodies but no paraneoplastic symptoms often have a better prognosis, indicating that localized breaches in self-tolerance, mediated by TLS in the TME, serve as a controlled mechanism for combating malignancy.

A break in localized self-tolerance may be the defining trigger for paraneoplastic syndromes. This could explain the high specificity but low sensitivity of autoantibody markers; only a subset of autoantibody-positive patients, presumably those with T cell extravasation into the bloodstream due to extended inflammation, develop paraneoplastic features. Tumor-associated TLS are linked with better prognosis and higher immune infiltration, underscoring their role in cancer immunity. Treatment with ICIs can also facilitate the development of autoimmunity through mechanisms that are often independent of autoantibodies.

## 5 Conclusion

The roles of autoantibodies and T cell autoreactivity in cancer-related autoimmunity remain incompletely understood, as preclinical and clinical data are still limited. A deeper understanding of AMPS could help us clarify the intersection of TLS biology, immune tolerance, and cancer immunity. Resolving how localized immune responses within the tumor microenvironment contribute to either paraneoplastic syndromes or effective tumor resolution may uncover novel therapeutic targets and biomarkers. The similarities between AMPS and ICI-induced irAEs evidence the complex balance between antitumor activity and autoimmunity, highlighting the need for personalized approaches to cancer immunotherapy. Future research focused on unraveling the mechanisms underlying TLS formation, T cell migration, and systemic immune tolerance breaks, will enable a deeper understanding of these processes and will improve both cancer treatment and the management of autoimmune complications.
